# Toxicity profile of combined immune checkpoint inhibitors and thoracic radiotherapy in esophageal cancer: A meta-analysis and systematic review

**DOI:** 10.3389/fimmu.2022.1039020

**Published:** 2022-11-10

**Authors:** Tongzhen Xu, Yunsong Liu, Xiaotong Lu, Jun Liang

**Affiliations:** ^1^ Department of Radiotherapy Oncology, National Cancer Center/National Clinical Research Center for Cancer/Cancer Hospital, Chinese Academy of Medical Sciences and Peking Union Medical College, Beijing, China; ^2^ Department of Radiotherapy Oncology, National Cancer Center/National Clinical Research Center for Cancer/Cancer Hospital & Shenzhen Hospital, Chinese Academy of Medical Sciences and Peking Union Medical College, Shenzhen, China

**Keywords:** esophageal cancer, safety, toxicity profile, immune checkpoint inhibitors, thoracic radiotherapy, systemic analysis

## Abstract

**Background:**

Therapies based on the combination of immune checkpoint inhibitors (ICIs) and thoracic radiotherapy (TRT) are transforming the treatment landscape of esophageal cancer. Nevertheless, the available data on adverse events (AEs) mainly stemmed from several prospective clinical trials and retrospective studies, in which, AE data are often handled and reported with less rigor than the primary beneficial outcomes of the study. Thus, we conducted a systematic review to investigate the toxicity spectrum of these novel regimens.

**Method:**

We searched for all prospective clinical trials investigating the role of ICIs combined with TRT published between January 2010 and August 2022. Study articles and conference proceedings involving esophageal cancers and reporting the overall incidence or details of treatment-related AEs (trAEs) were synthesized to determine the toxicity profile of combination treatment. We compared trAEs between cancer type, programmed cell death 1 (PD-1) and programmed cell death ligand 1 (PD-L1) inhibitors, and between sequential and concurrent administration of ICIs and TRT to identify potentially high-risk patients.

**Results:**

We obtained toxicity data from 14 clinical trials involving 863 patients. The pooled overall incidence was 88.97% for any-grade trAEs and 18.48% for high-grade trAEs. The three most frequent non-hematologic any-grade trAEs were reactive cutaneous capillary endothelial proliferation (RCCEP, 63.80%), esophagitis (51.54%), and fatigue (33.63%). Meanwhile, RCCEP (15.69%) was the most common non-hematologic high-grade trAE, followed by nausea (4.91%) and anorexia (3.81%). The occurrence rates of any-grade and high-grade pneumonitis were 10.82% and 0.66%, respectively. In subgroup analysis, the toxicity profiles of PD-1 and PD-L1 inhibitors were mostly similar, except for any-grade pneumonitis (15.20% vs 4.88%, p=0.03) and high-grade leukopenia (6.25% vs 59.09%, p=0.00). In addition, concurrent treatment seemed to have a higher incidence of any-grade trAEs (95.20% vs 70.85%, p=0.03) compared with sequential treatment. ESCC seems to have higher incidence of any-grade hypothyroidism (22.55% vs 8.96%, p=0.049) compared to EAC.

**Conclusion:**

Our study is the first systematic review to provide a toxicity profile of trAEs in esophageal cancer patients who received ICIs combined with TRT. Most AEs of this combination treatment are tolerable, although the incidence of any-grade trAEs was higher in the concurrent group. The difference in any-grade pneumonitis between PD-1 and PD-L1 inhibitor groups needs further validation in a large clinical trial.

## Introduction

Esophageal cancer is the seventh most common malignancy and the sixth leading cause of cancer-related mortality worldwide (1). Its histological subtypes mainly comprise esophageal squamous cell carcinoma (ESCC) and esophageal adenocarcinoma (EAC) and, its prognosis depends on the area involved, with estimated 5-year overall survival (OS) rates ranging from about 20% to 40% (2–4). Normally, radical resection is the first-line recommendation for early-stage tumors and while definitive chemoradiation is applied for inoperable locally advanced tumors. Nevertheless, despite ongoing development in surgical techniques and the optimization of chemoradiotherapy regimens, the OS benefits are still unable to meet clinicians’ expectations.

The advent of immune checkpoint inhibitors (ICIs), which include programmed cell death 1/programmed cell death ligand 1 (PD-1/PD-L1) and cytotoxic T-lymphocyte- associated antigen 4 (CTLA-4), has broadened the horizon for the treatment of various solid tumors. Nevertheless, preliminary data from clinical trials involving single-agent pembrolizumab or nivolumab in metastatic gastroesophageal cancer found response rates of just 22% to 27% in patients with PD-L1-positive tumors, impeding their further application and popularization (5, 6). Research into multimodal treatments incorporating TRT and ICIs has long been promoted in esophageal cancer (7–9). The CheckMate 577 trial indicated that the administration of adjuvant nivolumab, compared with placebo, provided a longer disease-free survival (22.4 vs. 11.0 months, p < 0.001) in esophageal and gastroesophageal cancer patients who had received neoadjuvant chemoradiotherapy followed by radical surgery (10). In addition, several clinical trials conducted in recent years also demonstrated the feasibility of combined of TRT and ICIs in (neo)adjuvant and maintenance settings of esophageal and gastroesophageal cancer (11–13).

The synergistic effects of TRT and ICIs have implications for both cancer control and toxicity risks in nonmalignant tissues. The rate of autoimmune-like disorders and even fatal adverse events (AEs) may rise (14), with potentially increased risks when they are combined with other agents (15). While the potential capability of this combination modality has fallen under the spot-light, the frequency and spectrum of treatment-related AEs (trAEs) during the procedure are yet to be detailed in esophageal cancer, with most toxicity data coming from individual clinical trial reports. Given the expected increased application of this combination therapy to esophageal cancer patients in the immediate future (16), treating physicians must comprehensively understand the spectrum and severity of the toxicities associated with these therapies, in turn shedding light on their clinical application and on the design of prospective trials. Hence, we conducted a systematic review focused on prospective clinical trials evaluating the AEs of the combination of TRT with ICIs in the field of esophageal cancer.

## Materials and methods

### Study search and inclusion criteria

This work was performed according to the Preferred Reported Items for Systematic Reviews and Meta-analyses (PRISMA) statement (17). The study selection and data extraction were performed independently by two authors (X.T. and L.Y.). Discrepancies were adjudicated by a third reviewer (L.J.) and resolved by consensus. The inclusion criteria for the literature search were defined using the Population, Intervention, Control, Outcome, Study Design (PICOS) framework (18, 19). Medical literature, including clinical trials, clinical studies, comparative studies, and multicenter studies, published in English up until July, 2022 was searched in PubMed, Embase, Scopus, Medline In Process, and Cochrane Library, using the following terms in combination with Boolean operators (AND, OR, NOT): esophageal cancer, radiotherapy or chemoradiotherapy, immune checkpoint inhibitors, and clinical trials. The full search strategy and results are summarized in **Supplementary Table 1**. Clinical trials meeting the following inclusion criteria were considered: (1) patients with histologically confirmed ESCC or EAC; (2) patients receiving combination ICI and TRT treatment; (3) clinical trials reporting the overall incidence of trAE profiles; and (4) studies published in English. Retrospective studies were excluded to minimize the risk of bias. Abstracts and presentations were also reviewed to identify relevant clinical trials from major conference proceedings between 2012 and 2022, including the American Society of Clinical Oncology, European Society of Medical Oncology, American Association for Cancer Research, and American Society for Radiation Oncology Annual Meeting. If multiple publications reporting on the same study population were identified, the article with the most up-to-date and/or comprehensive AE data was selected. We also performed a manual secondary search of all bibliographies from the final selected articles so as not to possibly miss eligible studies.

### Data extraction and statistical analysis

The following data were obtained from each included study: basic information (trial identifier, first author, and publication year), study methods (trial phase, study design, and enrollment), participants (age, sex, histology, clinical stage, drug name and type as well as dose and administration cycles, line of therapy, patterns of ICI and TRT combinations, and radiation dose and segmentation), outcomes (number of patients with at least one [any-grade or grade ≥ 3] trAE, number of patients who discontinued the regimen due to trAEs, number of treatment-related deaths and their causes, and the occurrence of trAEs recorded by at least three studies). The trAEs of interest included toxicities affecting the hematological system, skin system, gastrointestinal tract, respiratory system, and endocrine system. The AE terminology was coded according to the Medical Dictionary for Regulatory Activities, and the severity was graded according to the Common Terminology Criteria for Adverse Events. AEs described as immune-related AEs (irAEs) or selected AEs suspected to be potential irAEs were also extracted as trAEs in the present study. Methodological Index for Non-randomized Studies (MINORS) and Newcastle-Ottawa -Scale (NOS) evaluations were performed to assess the quality of the included studies (20, 21).

A meta-analysis of proportions was conducted using R version 4.2.1 (R Foundation for Statistical Computing). In anticipation of marked heterogeneity, the meta-analysis of outcomes was calculated with a random-effects restricted maximum-likelihood model, *via* the meta::metaprop function (22). Because of the inconsistency of the recorded trAEs among clinical trials, the information on trAEs in each study was extracted and the incidence of each trAE documented by at least three studies was finally pooled. The pooled incidence with its 95% confidence interval (CI) was estimated using the generalized linear mixed model, which implicitly uses the logit transformation (23). This model could be used to calculate the pooled incidence of rare event without assuming an approximate normal within-study likelihood and treating the standard errors as known as the traditional approach, the summary measures.

The inconsistency index (I^2^) and Cochrane chi-squared test were calculated to measure heterogeneity (24). The cut-offs 30.0%, 50.0%, and 75.0% denoted moderate, substantial, and considerable heterogeneity, respectively, as recommended by the guidance on the interpretation of heterogeneity scores in the Cochrane Handbook (25). Subgroup analyses of AE incidences and profiles were performed according to cancer type (ESCC/EAC), ICI agent types (PD-1/PD-L1) as well as the sequential and concurrent administration of ICIs and TRT. The probability of publication bias was assessed with the Egger’s linear regression test (26) and with the visual inspection of funnel plots for asymmetry, *via* the meta::metabias and meta::funnel functions, respectively. The non-parametric “trim-and-fill” method was performed to minimize the influence of publication bias on the results of existing publication bias, *via* the meta::trimfill function. A p value of < 0.05 was considered statistically significant.

## Results

### Eligible studies and baseline characteristics

**Figure 1:**
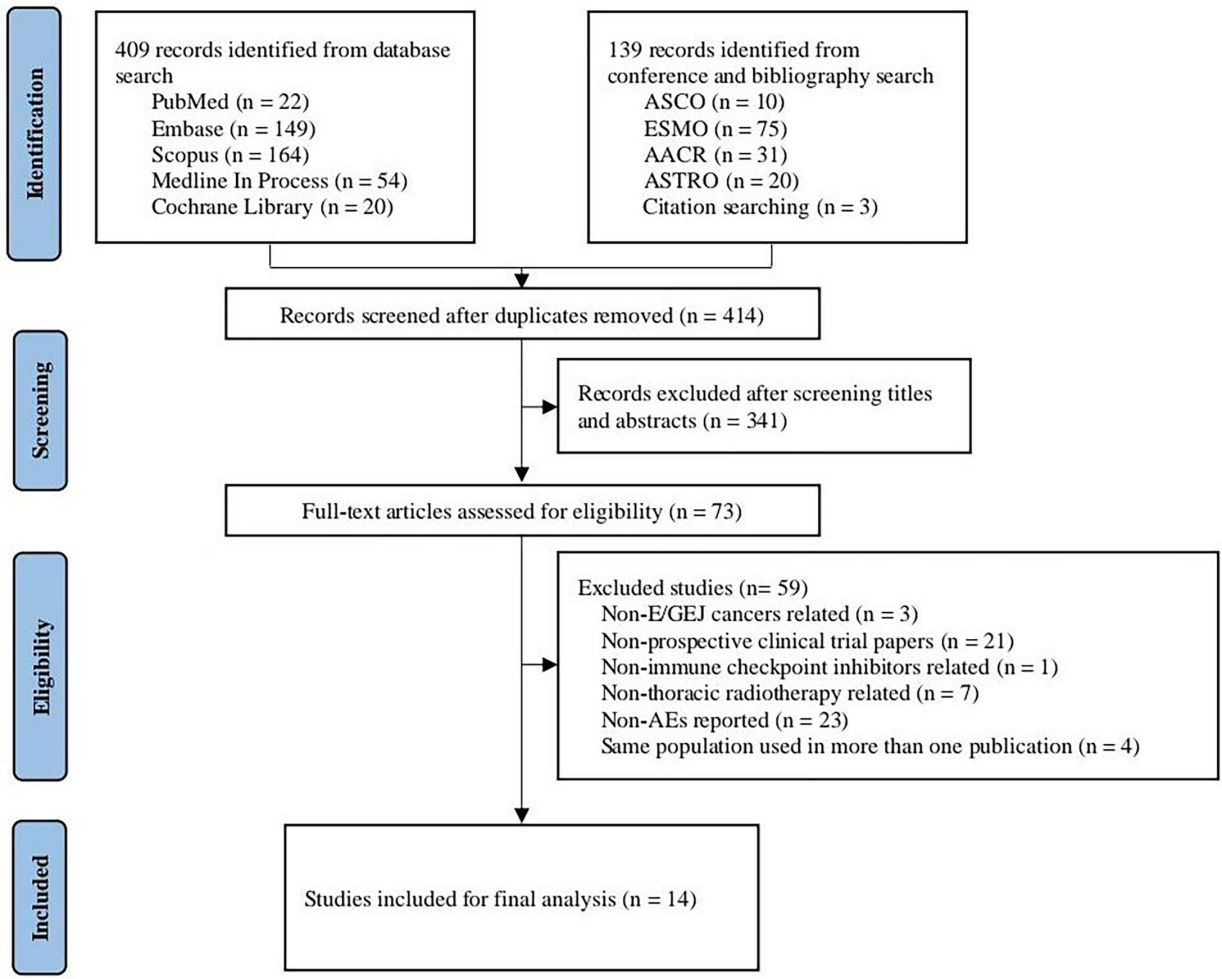
Diagram of study selection process. AEs, adverse events.

The combined systematic search strategy identified 548 records, of which, 134 duplicates were excluded; 73 records were ultimately deemed eligible for full-text screening. According to the inclusion criteria, 14 clinical trials were finally included in the analysis (10–12, 27–37). Among them, a total of 863 patients with esophageal cancer were eligible for quantitative analysis of trAE incidence, with the sample size sizes in these studies ranging from 11 to 532 participants. The main characteristics of the included studies are presented in **Supplementary Table 2** (baseline information) and **Supplementary Table 3** (safety information). The flow diagram of study selection is illustrated in **Figure 1**. Risk of bias assessments are summarized in **Supplementary Tables 4** using NOS and in **Supplementary Table 5** using MINORS.

The NOS of the included studies ranged from 6 to 9, while the MINORS ranged from 12 to 24. There were six phase I trials, seven phase II trials, and one phase III trial. Patients included in ten studies were all identified pathologically with esophageal squamous cell carcinoma, while at least 70% patients included in the other four studies were identified with esophageal adenocarcinoma. Notably, PD-1 inhibitors were used in 10 trials and PD-L1 inhibitors in four. Sequential administration of ICIs and radiotherapy was performed in five trials, while concurrent therapy was conducted in nine. The duration of ICI administration ranged from 2 months to 2 years. Surgery was performed in nine trials, comprising neoadjuvant ICI and TRT in six trials and consolidation ICI therapy in three.

Among the remaining five trials involving nonsurgical patients, two involved concurrent definitive TRT with ICIs and ICI consolidation, one involved received concurrent definitive TRT with ICIs, one involved definitive TRT plus sequential ICIs, and one involved concurrent palliative TRT with ICIs. In addition, two clinical trials involved patients with chemotherapy intolerance who received only radiotherapy plus ICIs, whereas others conducted chemoradiotherapy plus ICIs. TRT combined with ICI was administered in five trials, and was concurrent in four and sequential in one.

### Studies evaluating the incidence of trAEs

**Figure 2:**
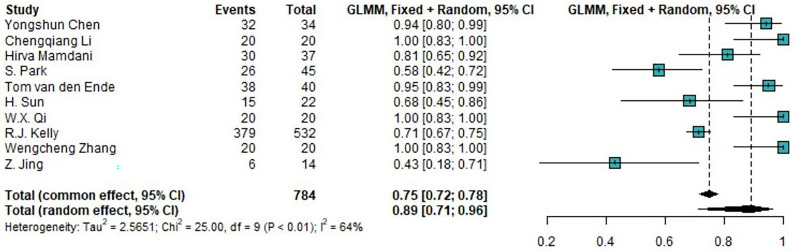
Forest plot of the incidence of any-grade treatment-related AEs. AEs, adverse events; GLMM, generalized linear mixed model.

**Figure 3:**
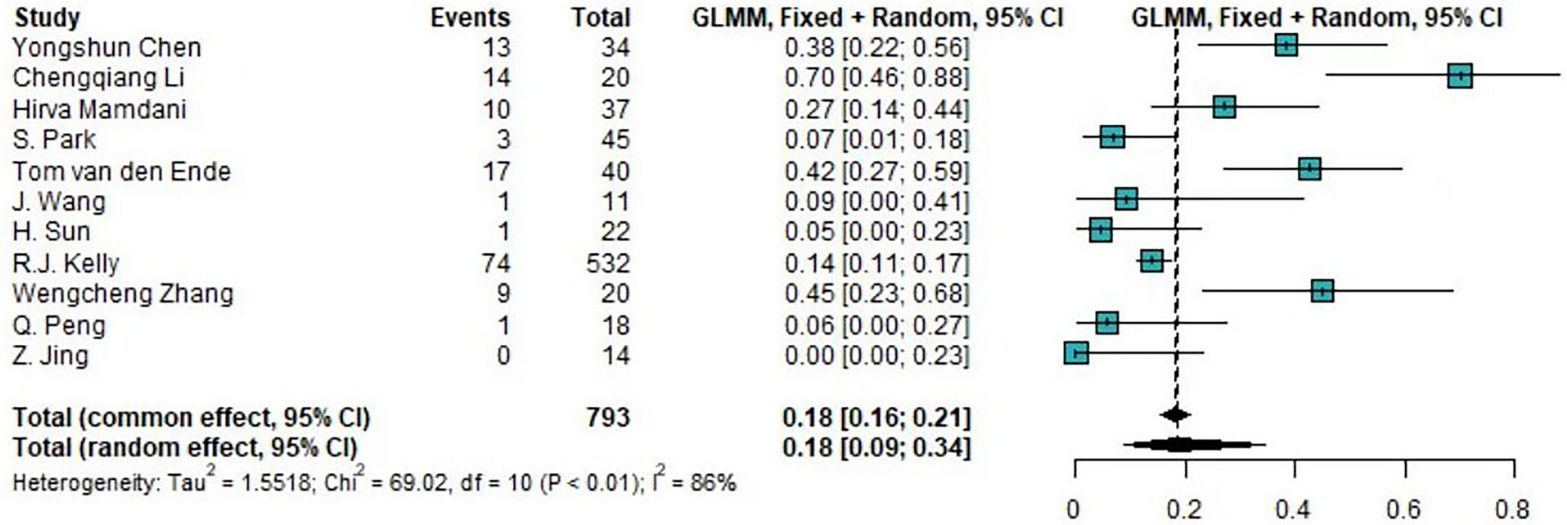
Forest plot of the incidence of high-grade treatment-related AEs. AEs, adverse events; GLMM, generalized linear mixed model.

Of the 14 clinical trials examining the combination of TRT and ICIs, we synthesized 10 trials (76.9%) reporting the incidence of any-grade trAEs and 11 trials (84.6%) reporting the incidence of high-grade trAEs. The pooled overall incidences was were 88.97% (95%CI 71.22%-96.34%) for any-grade trAEs and 18.48% (95%CI 8.90%-34.46%) for high-grade trAEs. Moreover, the pooled incidence of the discontinuation of ICIs due to trAEs was 8.24% (95%CI 6.52%-10.36%). The forest plots of any-grade trAEs and high-grade trAEs are shown in **Figure 2** and **Figure 3**, respectively.

Among any-grade trAEs, the most common was leukopenia (83.87%, 95%CI 34.76%-98.07%). The next most common was anemia (78.25%, 95%CI 66.03%-86.93%), after the use of “trim-and-fill” analysis to address its significant publication bias (p=0.00). Only two studies reported the incidence of any-grade lymphopenia, in which patients all developed any-grade lymphopenia (29, 35). The most common non-hematologic any-grade toxicity was reactive cutaneous capillary endothelial proliferation (RCCEP), with an incidence of 63.80% (95%CI 43.95%-79.84%), followed by esophagitis (51.54%, 95%CI 37.67%-65.18%) and fatigue (33.63%, 95%CI 13.82%-61.55%). Besides any-grade anemia, any-grade dermatitis (p=0.00) and any-grade elevated alanine transaminase (ALT, p=0.05), both of which had a positive publication bias with the Egger’s test, were modified by the “trim-and-fill” method to have incidences of 15% (95%CI 7.5%-27.75%) and 20.81% (95%CI 14.17%-29.50%). The funnel plots of any-grade dermatitis and elevated ALT after “trim-and-fill” analysis are shown in **Supplementary Figure 1**. Besides hematologic toxicity and RCCEP, trAEs related to the gastrointestinal tract were the most frequently observed trAEs, and included anorexia (20.84%), diarrhea (15.42%), and nausea (14.34%). In addition, endocrine system trAEs were also common, including hypothyroidism (12.26%) and hyperthyroidism (6.88%). The incidences of elevated aspartate aminotransferase (AST), pneumonitis, pruritus, skin rash, and arthralgia were 12.89%, 10.82%, 10.32%, 10.03%, and 6.19%, respectively (**Table 1**).

**Table 1:** Incidences of any-grade AEs recorded by at least three studies.

Adverse events	Pooled incidence	95%CI	Recorded studies, No.	Heterogeneity	χ^2^	Egger test p
All-grade AEs	88.97%	71.22-96.34	10	64.0%	72.81	0.11
Leukopenia	83.87%	34.76-98.07	6	62.5%	59.81	0.10
Anemia	78.25%	66.03-86.93	3	0.0%	0.20	**0.00**
RCCEP	63.80%	43.95-79.84	3	56.2%	5.33	0.48
Esophagitis	51.54%	37.67-65.18	7	61.3%	18.91	0.88
Fatigue	33.63%	13.82-61.55	8	89.7%	138.53	0.16
Anorexia	20.84%	4.02-62.36	3	82.4%	28.05	0.27
Elevated ALT	20.81%	14.17-29.50	6	44.9%	14.51	**0.05**
Diarrhea	15.42%	12.92-18.30	6	0.0%	25.56	0.29
Dermatitis	15.00%	7.50-27.75	3	28.1%	5.56	**0.00**
Nausea	14.34%	6.18-29.83	8	88.0%	67.36	0.58
Elevated AST	12.89%	7.07-22.34	6	83.6%	27.08	0.21
Hypothyroidism	12.26%	5.05-26.87	5	87.9%	30.53	0.82
Pneumonitis	10.82%	6.02-18.69	8	44.3%	14.33	0.06
Pruritus	10.32%	8.11-13.04	3	0.0%	1.32	0.66
Skin rash	10.03%	7.97-12.55	5	0.0%	2.65	0.78
Hyperthyroidism	6.88%	5.14-9.15	4	0.0%	0.50	0.08
Arthralgia	6.19%	4.54-8.39	3	67.9%	5.10	0.72

The bold values mean that these AEs have significant publication bias in the Egger test, and the pooled incidence of there AEs was calculated by using the "trim-and-fill" method to addressing this publication bias.

Among high-grade trAEs, the Egger’s test indicated publication bias for lymphopenia, leukopenia, RCCEP, nausea, anemia, and arthralgia, and the “trim-and-fill” method was applied to address the bias and calculate the pooled incidence. The funnel plots of high-grade toxicities after the “trim-and-fill” method are shown in **Supplementary Figure 2**. Lymphopenia was the most common high-grade trAE, with an incidence of 65.57% (95%CI 51.02%-77.69%), followed by leukopenia (21.93%, 95%CI 7.51%-49.31%). The incidence of high-grade anemia was 2.44% (95%CI 0.79%-7.29%). RCCEP (15.69%, 95%CI 8.29%-27.70%) was the most common non-hematologic high-grade trAEs, followed by nausea (4.91%, 95%CI 1.77%-12.89%) and anorexia (3.81%, 95%CI 1.44%-9.71%). High-grade esophagitis and fatigue occurrences were 2.6% (95%CI 0.98%-6.71%) and 0.41% (95%CI 0.13%-1.27%), respectively. In addition, the incidences of high-grade elevated ALT, elevated AST, hepatitis, skin rash, pneumonitis, diarrhea, and arthralgia were 1.44%, 1.16%, 0.98%, 0.90%, 0.66%, 0.43%, and 0.19%, respectively. The incidence of grade 5 AEs was 0.36% (95%CI 0.12%-1.11%) (**Table 2**).

**Table 2:** Incidences of high-grade AEs recorded by at least three studies.

Adverse events	Pooled incidence	95%CI	Recorded studies, No.	Heterogeneity	χ^2^	Egger test p
High-grade AEs	18.48%	8.90-34.46	11	85.5%	79.75	0.57
Lymphopenia	65.57%	51.02-77.69	3	66.9%	6.05	**0.00**
Leukopenia	21.93%	7.51-49.31	6	77.8%	31.52	**0.01**
RCCEP	15.69%	8.29-27.70	4	43.0%	8.77	**0.01**
Nausea	4.91%	1.77-12.89	8	53.1%	23.47	**0.05**
Anorexia	3.81%	1.44-9.71	3	0.0%	7.98	0.15
Esophagitis	2.60%	0.98-6.71	7	0.0%	17.08	0.88
Anemia	2.44%	0.79-7.29	4	0.0%	0.00	**0.00**
Elevated ALT	1.44%	0.36-5.57	5	0.0%	3.61	0.18
Elevated AST	1.16%	0.24-5.44	5	66.3%	12.9	0.79
Hepatitis	0.98%	0.14-6.63	3	0.0%	2.05	0.53
Skin rash	0.90%	0.40-1.98	5	17.2%	5.52	0.57
Pneumonitis	0.66%	0.20-1.38	9	0.0%	4.88	0.21
Diarrhea	0.43%	0.14-1.33	6	0.0%	3.13	0.10
Fatigue	0.41%	0.13-1.27	8	0.0%	12.05	0.29
Grade 5 AEs	0.36%	0.12-1.11	13	0.0%	14.53	0.12
Arthralgia	0.19%	0.05-0.67	3	41.7%	6.86	**0.04**

The bold values mean that these AEs have significant publication bias in the Egger test, and the pooled incidence of there AEs was calculated by using the "trim-and-fill" method to addressing this publication bias.

### trAE incidence by ICI agent type

A comparison of trAEs between PD-1 and PD-L1 inhibitors combined with TRT is shown in **Table 3**. A pooled subgroup analysis was not possible for RCCEP, leukopenia, lymphopenia, and anemia, because clinical trials reporting these trAEs all just used PD-1 inhibitors alone, except for high-grade leukopenia. The incidences of any-grade and high-grade trAEs were 93.14% (95%CI 64.91%-99.01%) and 16.49% (95%CI 5.87%-38.48%) in the PD-1 inhibitor group and 81.74% (95%CI 56.85%-93.83%) and 22.06% (95%CI 8.72%-45.63%) in the PD-L1 inhibitor group. There were no significant differences in any-grade and high-grade toxicities between the PD-1 and PD-L1 inhibitor groups, except for any-grade pneumonitis (15.20% vs 4.88%, p=0.03) and high-grade leukopenia (6.25% vs 59.09%, p=0.00). There was no significant difference in high-grade pneumonitis (0.62% vs 2.44%, p=0.11). The incidence of high-grade fatigue was nonsignificantly higher in the PD-L1 inhibitor group than in the PD-1 inhibitor group (1.64% vs 0.17%, p=0.06).

**Table 3:** Differences in the incidence of AEs with PD-1 vs PD-L1 inhibitors combined with TRT.

	PD-1 Incidence (%) (95%CI)	PD-L1 Incidence (%) (95%CI)	p value
**All-grade AEs**	93.14 (64.91-99.01)	81.74 (56.85-93.83)	0.35
Esophagitis	49.68 (33.46-65.97)	60.00 (44.35-73.85)	0.38
Anorexia	45.00 (25.32-66.38)	12.09 (1.01-64.91)	0.20
Fatigue	29.93 (17.08-46.98)	40.24 (3.38-92.83)	0.77
Diarrhea	15.73 (12.97-18.95)	13.93 (8.84-21.28)	0.62
Elevated ALT	15.56 (9.43-24.57)	14.29 (8.09-23.99)	0.99
Pneumonitis	15.20 (9.25-23.95)	4.88 (1.84-12.29)	**0.03**
Nausea	14.90 (7.78-26.65)	9.50 (0.55-66.74)	0.74
Pruritus	9.93 (7.70-12.71)	16.22 (7.47-31.69)	0.23
Skin rash	9.89 (7.65-12.69)	10.66 (6.29-17.49)	0.80
Hypothyroidism	9.59 (7.42-12.31)	12.97 (1.38-61.30)	0.78
Elevated AST	7.31 (5.48-9.68)	14.29 (8.09-23.99)	0.82
Hyperthyroidism	6.63 (4.82-9.06)	8.54 (4.12-16.84)	0.53
Arthralgia	5.64 (3.97-7.95)	9.37 (3.76-21.49)	0.30
**High-grade AEs**	16.49 (5.87-38.48)	22.06 (8.72-45.63)	0.66
Leukopenia	6.25 (2.83-13.22)	59.09 (38.17-77.16)	**0.00**
Elevated ALT	1.61 (0.23-10.57)	1.30 (0.18-8.64)	0.88
Skin rash	0.73 (0.28-1.94)	1.64 (0.41-6.32	0.35
Pneumonitis	0.62 (0.23-1.65)	2.44 (0.61-9.23)	0.11
Elevated AST	0.52 (0.17-1.61)	2.83 (0.27-23.65)	0.20
Diarrhea	0.35 (0.09-1.39)	0.82 (0.12-5.58)	0.49
Grade 5 AEs	0.23 (0.01-4.05)	0.69 (0.10-4.76)	0.53
Arthralgia	0.19 (0.00-0.74)	0.00 (0.00-1.17)	0.46
Fatigue	0.17 (0.02-1.16)	1.64 (0.41-6.32)	0.06

The bold values mean that there are significant statistical differences in subgroup analysis of these AEs.

### trAE incidence by the concurrent and sequential administration of ICIs and TRT

A comparison of trAEs between the concurrent and sequential administration of ICIs and TRT is shown in **Table 4**. A pooled subgroup analysis was not possible for esophagitis, arthralgia, hyperthyroidism, dermatitis, leukopenia, and anemia, because clinical trials reporting these trAEs could not be divided as they were all separate concurrent or sequential studies. The incidences of any-grade and high-grade trAEs were 95.20% (95%CI 73.75%-99.29%) and 21.59% (95%CI 7.00%-50.18%) in the concurrent group, and 70.85% (95%CI 67.13%-74.31%) and 14.08% (95%CI 11.57%-17.03%) in the sequential group. There was a significant difference between the concurrent and sequential groups in any-grade trAEs (p=0.03). In addition, the concurrent group seemed to show significantly increased incidences of any-grade fatigue (52.04% vs 14.91%, p=0.02), anorexia (43.33% vs 2.22%, p=0.00), nausea (29.51% vs 7.52%, p=0.01), and elevated AST (17.07% vs 5.80%, p=0.00).

**Table 4:** Differences in the incidence of AEs with concurrent vs sequential ICIs combined with TRT.

	Sequential Incidence (%) (95%CI)	Concurrent Incidence (%) (95%CI)	p value
**All-grade AEs**	70.85 (67.13-74.31)	95.20 (73.75-99.29)	**0.04**
RCCEP	54.55 (26.81-79.72)	67.70 (40.32-86.67)	0.51
Fatigue	14.91 (7.46-27.56)	52.04 (21.11-81.49)	**0.03**
Anorexia	2.22 (0.31-14.16)	43.33 (31.46-56.03)	**0.00**
Nausea	7.52 (3.05-17.40)	29.51 (14.78-50.28)	**0.01**
Hypothyroidism	11.50 (7.27-17.74)	15.00 (4.92-37.58)	0.65
Elevated AST	5.80 (4.15-8.05)	19.92 (13.08-29.15)	**0.00**
Elevated ALT	10.81 (4.12-25.49)	15.81 (9.66-24.79)	0.47
Pruritus	10.37 (8.12-13.15)	9.09 (2.28-29.96)	0.85
Skin rash	9.93 (7.81-12.56)	11.11 (5.08-22.60)	0.78
Diarrhea	15.47 (12.82-18.55)	15.00 (8.72-24.58)	0.91
Pneumonitis	9.11 (2.31-29.79)	13.00 (7.70-21.11)	0.61
**High-grade AEs**	14.08 (11.57-17.03)	21.59 (7.00-50.18)	0.44
Leukopenia	59.09 (38.17-77.16)	6.25 (2.83-13.22)	**0.00**
Elevated AST	1.54 (0.17-12.70)	1.22 (0.17-8.15)	0.88
Pneumonitis	0.89 (0.30-1.78)	0.00 (0.00-0.96)	0.08
Elevated ALT	2.70 (0.38-16.85)	0.98 (0.14-6.63)	0.47
Skin rash	0.65 (0.24-1.72)	3.70 (0.93-13.64)	**0.04**
Fatigue	0.16 (0.02-1.15)	1.75 (0.44-6.74)	**0.051**

The bold values mean that there are significant statistical differences in subgroup analysis of these AEs.

In terms of high-grade trAEs, the incidences of leukopenia (6.25% vs 59.09%, p=0.00) and skin rash (0.65% vs 3.70%, p=0.04) were higher in the sequential group than in the concurrent group. The incidence of high-grade fatigue was nonsignificantly higher in the concurrent group (1.75% vs 0.16%, p=0.051).

### trAE incidence by cancer type

A comparison of trAEs between ESCC and EAC is shown in **Table 5**. We compared the toxicity profile between studies which only included ESCC patients and studies which mainly included EAC patients (at least 70%). A pooled subgroup analysis was not possible for RCCEP, dermatitis, leukopenia, anemia and lymphopenia because clinical trials reporting these trAEs could not be divided into different cancer type groups. The incidences of any-grade and high-grade trAEs were 92.96% (95%CI 61.56%-99.09%) and 14.41% (95%CI 4.62%-36.91%) in the ESCC group, and 60.00% (95%CI 44.35%-73.85%) and 24.75% (95%CI 13.43%-41.09%) in the EAC group. There were no significant differences in any-grade and high-grade toxicities between the ESCC and EAC groups, except for any-grade hypothyroidism (22.55% vs 8.96%, p=0.049) and high-grade leukopenia (6.25% vs 59.09%, p=0.00).

**Table 5:** Differences in the incidence of AEs with ESCC vs EAC groups.

	ESCC Incidence (%) (95%CI)	EAC (at least 70%) Incidence (%) (95%CI)	p value
**All-grade AEs**	92.96 (61.56-99.09)	83.31 (66.21-92.71)	0.41
Esophagitis	49.68 (33.46-65.97)	60.00 (44.35-73.85)	0.38
Fatigue	25.16 (10.22-49.83)	51.80 (9.65-91.54)	0.37
Hypothyroidism	22.55 (9.34-45.15)	8.96 (6.88-11.60)	**0.049**
Elevated AST	20.26 (10.22-36.19)	8.84 (4.53-16.53)	0.08
Skin rash	15.25 (8.13-26.79)	9.52 (7.43-12.12)	0.17
Elevated ALT	14.19 (6.89-26.98)	14.29 (8.09-23.99)	0.99
Pneumonitis	12.89 (7.70-20.80)	2.70 (0.38-16.85)	0.11
Anorexia	12.23 (0.95-66.87)	42.50 (28.31-58.04)	0.23
Nausea	10.05 (2.70-31.04)	20.94 (7.39-46.76)	0.36
Pruritus	9.09 (2.28-29.96)	10.37 (8.12-13.15)	0.85
Hyperthyroidism	8.93 (3.77-19.72)	6.68 (4.90-9.05)	0.53
Arthralgia	4.44 (1.11-16.11)	8.01 (3.57-16.96)	0.46
Diarrhea	1.12 (0.02-34.05)	17.24 (14.44-20.45)	0.14
**High-grade AEs**	14.41 (4.62-36.91)	24.75 (13.43-41.09)	0.37
Leukopenia	6.25 (2.83-13.22)	59.09 (38.17-77.16)	**0.00**
Elevated AST	2.38 (0.33-15.06)	0.99 (0.12-7.64)	0.55
Fatigue	1.68 (0.42-6.47)	0.12 (0.00-7.80)	0.24
Elevated ALT	1.61 (0.23-10.57)	1.30 (0.18-8.64)	0.88
Grade 5 AEs	0.98 (0.25-3.83)	0.10 (0.00-6.72)	0.33
Pneumonitis	0.64 (0.09-4.41)	0.88 (0.37-2.09)	0.77

The bold values mean that there are significant statistical differences in subgroup analysis of these AEs.

## Discussion

Ours study is the first systematic review to provide a relatively comprehensive toxicity profile of trAEs in esophageal cancer patients who received ICIs combined with TRT. We reached the following conclusions: (1) a high proportion of patients experienced at least one any-grade trAE, with a marked variation between the sequential and concurrent administration of TRT and ICIs; (2) the prevalence of grade ≥ 3 treatment-related toxicity was substantial, with approximately one-tenth of patients discontinuing ICI administration due to trAEs, and treatment-related mortality was only 0.36%; and (3) by pooling the incidence of trAEs in esophageal cancer in an approaching era marked by the inevitable growth in the combination of ICIs with TRT in the treatment practice for esophageal cancer, we are the first to find that dermatological and gastrointestinal reactions are the most common non-hematological toxicities.

Overall, our pooled analysis identified rates of 88.97% and 18.48% for any-grade trAEs and high-grade trAEs, respectively. The most frequent high-grade trAE was lymphopenia (65.57%), followed by leukopenia (21.93%), RCCEP (15.69%), nausea (4.91%), and anorexia (3.81%). This toxicity profile of combined TRT and ICIs was remarkably different from that of previous studies without the addition of ICIs. As is well known, definitive chemoradiotherapy was established decades ago as a curative alternative for inoperable patients with esophageal squamous cell carcinoma that confers a substantial improvement (38, 39). Treatment-related toxicity was not uniformly reported, with roughly 30%-60% of patients experiencing at least one high-grade AE. Of high-grade AEs, the most common hematotoxicity was leukopenia (10%-50%), followed by gastrointestinal toxicity (10%-25%) (40–43). Skin reaction, mostly radiation dermatitis, was uncommon at 8.9% (41). In the perioperative setting, neoadjuvant chemoradiotherapy followed by surgery has been proved to have greater local tumor control but similar OS when compared to definitive chemoradiation without surgery (44, 45) and to have a longer OS as well as and disease-free survival when compared to surgery alone (46). The incidences of grade ≥ 3 hematological and gastrointestinal toxicities were 10%-50% and 5%-10% in the neoadjuvant group. The frequencies of the other AEs were, in descending order, as follows: leukopenia (6%-48.8%), anorexia (2.2%-5%), vomiting (1%-4%), and esophagitis (1%-2.7%) (47, 48).

The incidences of blood and gastrointestinal toxicities in chemoradiation alone groups are similar to those of our data, indicating that the addition of ICIs to chemoradiation likely does not markedly increase the toxicity of these two treatments. In our results, the most common high-grade toxicity was lymphopenia. Korese et al. (49) determined that severe lymphopenia may lead to a worser prognosis in esophageal cancer patients treated with chemoradiation followed by surgery. Our results show that the concurrent group might develop an exacerbation of any-grade trAEs, mainly comprising fatigue (52.04% vs 14.91%, p=0.02), gastrointestinal tract disorders (anorexia, 43.33% vs 2.22%, [p=0.00]; nausea, 29.51% vs 7.52%, [p=0.01]), and hepatic dysfunction (elevated AST, 17.07% vs 5.80%, [p=0.00]). Thus, gastrointestinal toxicity should be monitored more carefully during concurrent TRT and ICIs.

RCCEP occurs on the skin surface, mainly on the surface of the head, face, and trunk. It is more likely to be considered immune-related, with incidences ranging from 8.9%-97.3% for grade 1 and 2 and 0% for grade ≥ 3 in ICI-related trials for various carcinomas (50–52). The evidence even suggests that the development of RCCEP may be positively associated with a longer OS and PFS (52). Our results show that the risk of high-grade RCCEP increases with combined TRT with ICIs. The addition of ICIs to chemoradiation may increase the presence of RCCEP, complicating the spectrum of dermatological toxicity, which is dominated by dermatitis induced by prior radiation (41, 53). Previous studies have suggested that radiotherapy-induced changes in proinflammatory cytokines and growth factors may contribute to surrounding normal tissue toxicities (54) and that immunotherapy may also promote vascular proliferation by releasing specific cytokines from immune cells (52). The synergistic effect may increase the probability of skin toxicity *via* the combination of TRT with ICIs.

The overall incidence of pneumonitis in chemoradiotherapy for esophageal cancer ranges from 10% to 15% (42, 43), with Chen et al. (42) reporting no presence of grade 3 or higher pneumonitis and Ji. et al. (43) identifying an incidence of 1.4% for high-grade pneumonitis. Previous studies illustrated that lung injury may be augmented by the combination of TRT with ICIs through the compound effect of tumor necrosis factor- (TNF), transforming growth factor-β (TGF-β), and other immune cytokines (55–59). This phenomenon conformed to the practice in the non-small cell lung cancer (NSCLC) setting, where grade 3 and 4 pneumonitis is observed at 4.4% and lethal pneumonitis is 0.8% with combined TRT and ICIs (60). In our study, 10% of patients experienced any-grade treatment-related pneumonitis and a markedly lower proportion (0.6%) encountered high-grade pneumonitis; there was no pneumonitis-related death. The incidence of any-grade pneumonitis was higher in the PD-1 inhibitor group than in the PD-L1 inhibitor group (15.20% vs 4.88%, p=0.03). However, there was no significant difference in high-grade pneumonitis between the two groups. These results differed from that of a previous study stating that PD-L1 inhibitors potentially carry a lower risk of pneumonitis (61, 62). The reasons underpinning this observation remain to be determined. We consider that this may have resulted from the inadequate inclusion of available full texts in our analysis. However, it is important to understand and investigate this situation in the esophageal cancer setting because such AEs may still deteriorate and require high-dose corticosteroids treatment, delaying patients’ definitive surgical treatment or other local treatment.

Hepatitis, thyroiditis, and colitis are the common autoimmune diseases of ICI monotherapy, with the incidences of 8.6%, 5.2%, and 3.8%, respectively (63). Only three clinical trials reported data on irAEs but they were inconsistent in their definitions (10, 32, 34), leading to an inadequate for pooled analysis of irAEs in our study. Therefore, we presented the pooled trAE data for hepatitis, thyroiditis, and colitis. First, the pooled incidences of treatment-related elevated ALT and AST were 20.81% and 12.89% for any-grade and 1.16% and 1.44% for high-grade, respectively (10–12, 29, 30, 33, 34, 37). This seems slightly higher than that of ICI monotherapy, but the role of concurrent chemotherapy during radiotherapy may have contributed to this increase in our systematic review. Kelly et al. (10) reported the presence of two patients, recorded specifically as serious hepatitis and requiring hospitalization, but the authors did not explain whether these events were treatment-related. Second, only one clinical trial (n=22) reported immune-related thyroiditis—two cases—and both were limited to grade 2 (32), although several clinical trials identified a treatment-related thyroid functional abnormality, leading to pooled incidences of any-grade hypothyroidism and hyperthyroidism of 12.26% and 6.88%, respectively (10, 12, 31, 33, 34). All treatment-related thyroid dysfunctions were mild without ≥ grade 3 events. It is noted that ESCC seems to have higher incidence of any-grade hypothyroidism in the combination treatment compared to EAC, which may be linked to the esophageal tumor’s locations. Esophageal cancer in the cervical and upper esophageal areas are mainly ESCC, while EAC occurs usually in the lower esophagus or the gastroesophageal junction. A lack of details on the irradiated site of the primary tumor prevents further investigation, because the thyroid dysfunction may be related to its irradiation when the tumor is located in cervical and upper esophageal areas. Thirdly, unlike the clear description of just two patients (out of 37) who developed possibly immune-related colitis by Mamdani et al. (34) and of three serious any-cause colitis (out of 532) requiring for hospitalization by Kelly et al. (10), most trials reported data on treatment-related diarrhea, one of the manifestations of colitis and other gastrointestinal tract AEs, with pooled incidence of 15.42% for any-grade and 0.43% for high-grade (10–12, 33–35). It should be noted that this apparent high frequency is likely due to a mixed diagnosis of different gastrointestinal toxicities, which often cause ill-defined and nonspecific symptoms. In addition, the studies included in our analysis did not provide detailed reasons for the discontinuation of ICI administration, preventing any further meaningful clinical conclusion and comparison with our data. This difference implies the need for the accurate monitoring of the corresponding organ function in clinical practice, with laboratory tests, ultrasonic examination, and imaging, to facilitate the identification of early AEs and promote timely intervention in these events.

This study has several limitations. First, this clinical trial-based meta-analysis limits the generalizability of our results to ordinary people in the real-world setting. Second, the relatively small number of eligible studies included in our analysis and the diagnosis and inconsistent recording of AEs performed by investigators without strict standardization somewhat hinder our further overview of trAEs and irAEs. Third, potential considerable heterogeneity and unobserved cofounders might prevent the interpretation of the overall incidence of various trAEs. Nevertheless, our systematic review of trAEs for combined TRT and ICIs can provide a relatively comprehensive insight into esophageal cancer for clinicians, promoting clinical vigilance and patient counseling. Further larger-scale, multicenter, randomized controlled trials and real-world studies are warranted to evaluate the safety of the combination of ICIs and TRT in patients with esophageal cancer.

## Conclusions

Our study is the first systematic review to provide a relatively comprehensive toxicity profile of trAEs in esophageal cancer patients who received ICIs combined with TRT. Most AEs of this combination treatment are tolerable, with the concurrent modality possibly having a higher incidence of any-grade trAEs than the sequential approach. Compared with PD-L1 inhibitors, PD-1 inhibitors might increase the incidence of any-grade pneumonitis. ESCC seems to have higher incidence of any-grade hypothyroidism compared to EAC. This finding needs further validation in larger clinical trials. These results indicate the importance of the early recognition of AE onset to facilitate efficient interventions that mitigate their severity and optimize outcomes in these patients.

## Data availability statement

The original contributions presented in the study are included in the article/**Supplementary Material**. Further inquiries can be directed to the corresponding author.

## Author contributions

Methodology, TX, YL, and JL. Software, TX, YL, XL. Validation, TX, YL, and JL. Formal analysis, TX and YL. Data curation, TX, YL, XL. Writing-original draft preparation, TX, YL and XL. Writing–review and editing, JL. Visualization, TX and YL. Project administration, TX and JL. Funding acquisition, JL. All authors contributed to the article and approved the submitted version.

## Funding

This work was supported by National Cancer Center/National Clinical Research Center for Cancer/Cancer Hospital & Shenzhen Hospital, Chinese Academy of Medical Sciences and Peking Union Medical College (SZ2020MS004).

## Conflict of interest

The authors declare that the research was conducted in the absence of any commercial or financial relationships that could be construed as a potential conflict of interest.

## Publisher’s note

All claims expressed in this article are solely those of the authors and do not necessarily represent those of their affiliated organizations, or those of the publisher, the editors and the reviewers. Any product that may be evaluated in this article, or claim that may be made by its manufacturer, is not guaranteed or endorsed by the publisher.
