# Corrigendum: Toxicity profile of combined immune checkpoint inhibitors and thoracic radiotherapy in esophageal cancer: A meta-analysis and systematic review

**DOI:** 10.3389/fimmu.2023.1193710

**Published:** 2023-04-11

**Authors:** Tongzhen Xu, Yunsong Liu, Xiaotong Lu, Jun Liang

**Affiliations:** ^1^ Department of Radiotherapy Oncology, National Cancer Center/National Clinical Research Center for Cancer/Cancer Hospital, Chinese Academy of Medical Sciences and Peking Union Medical College, Beijing, China; ^2^ Department of Radiotherapy Oncology, National Cancer Center/National Clinical Research Center for Cancer/Cancer Hospital & Shenzhen Hospital, Chinese Academy of Medical Sciences and Peking Union Medical College, Shenzhen, China

**Keywords:** esophageal cancer, safety, toxicity profile, immune checkpoint inhibitors, thoracic radiotherapy, systemic analysis

In the published article, there was an error. Two words were misspelled.

A correction has been made to **Conclusions**, Paragraph One. This sentence previously stated:

“Compared with PD-1 inhibitors, PD-L1 inhibitors might increase the incidence of any-grade pneumonitis.”

The corrected sentence appears below:

“Compared with PD-L1 inhibitors, PD-1 inhibitors might increase the incidence of any-grade pneumonitis.”

The authors apologize for this error and state that this does not change the scientific conclusions of the article in any way. The original article has been updated.

