# Supplementary material for: Toxicity profile of combined immune checkpoint inhibitors and thoracic radiotherapy in esophageal cancer: A meta-analysis and systematic review
Source: Front Immunol. 2022 Nov 10;13:1039020. doi: 10.3389/fimmu.2022.1039020 (PMC9685562; doi:10.3389/fimmu.2022.1039020)
Supplement: Supplementary file 1 [file DataSheet_1.pdf]

Supplementary Table 1. Search algorithm and results

Pubmed	Searching Strategy	Results
#1	("Esophageal Neoplasms"[Mesh]) OR (Esophageal Neoplasm[Title/Abstract]) OR (Neoplasm, Esophageal[Title/Abstract]) OR (Esophagus Neoplasm[Title/Abstract]) OR (Esophagus Neoplasms[Title/Abstract]) OR (Neoplasm, Esophagus[Title/Abstract]) OR (Neoplasms, Esophagus[Title/Abstract]) OR (Neoplasms, Esophageal[Title/Abstract]) OR (Cancer of Esophagus[Title/Abstract]) OR (Cancer of the Esophagus[Title/Abstract]) OR (Esophagus Cancer[Title/Abstract]) OR (Cancer, Esophagus[Title/Abstract]) OR (Cancers, Esophagus[Title/Abstract]) OR (Esophagus Cancers[Title/Abstract]) OR (Esophageal Cancer[Title/Abstract]) OR (Cancer, Esophageal[Title/Abstract]) OR (Cancers, Esophageal[Title/Abstract]) OR (Esophageal Cancers[Title/Abstract])	63822
#2	("Radiotherapy"[Mesh]) OR (Radiotherapies[Title/Abstract]) OR (Radiation Therapy[Title/Abstract]) OR (Radiation Therapies[Title/Abstract]) OR (Therapies, Radiation[Title/Abstract]) OR (Therapy, Radiation[Title/Abstract]) OR (Radiation Treatment[Title/Abstract]) OR (Radiation Treatments[Title/Abstract]) OR (Treatment, Radiation[Title/Abstract]) OR (Radiotherapy, Targeted[Title/Abstract]) OR (Radiotherapies, Targeted[Title/Abstract]) OR (Targeted Radiotherapies[Title/Abstract]) OR (Targeted Radiotherapy[Title/Abstract]) OR (Targeted Radiation Therapy[Title/Abstract]) OR (Radiation Therapies, Targeted[Title/Abstract]) OR (Targeted Radiation Therapies[Title/Abstract]) OR (Therapies, Targeted Radiation[Title/Abstract]) OR (Therapy, Targeted Radiation[Title/Abstract]) OR (Radiation Therapy, Targeted[Title/Abstract])	259631
#3	("Chemoradiotherapy"[Mesh]) OR (Chemoradiotherapies[Title/Abstract]) OR (Radiochemotherapy[Title/Abstract]) OR (Radiochemotherapies[Title/Abstract]) OR (Concurrent Chemoradiotherapy[Title/Abstract]) OR (Chemoradiotherapies, Concurrent[Title/Abstract]) OR (Chemoradiotherapy, Concurrent[Title/Abstract]) OR (Concurrent Chemoradiotherapies[Title/Abstract]) OR (Synchronous Chemoradiotherapy[Title/Abstract]) OR (Chemoradiotherapies, Synchronous[Title/Abstract]) OR (Chemoradiotherapy, Synchronous[Title/Abstract]) OR (Synchronous Chemoradiotherapies[Title/Abstract]) OR (Concurrent Radiochemotherapy[Title/Abstract]) OR (Concurrent Radiochemotherapies[Title/Abstract]) OR (Radiochemotherapies, Concurrent[Title/Abstract]) OR (Radiochemotherapy, Concurrent[Title/Abstract]) OR (Concomitant Chemoradiotherapy[Title/Abstract]) OR (Chemoradiotherapies, Concomitant[Title/Abstract]) OR (Chemoradiotherapy, Concomitant[Title/Abstract]) OR (Concomitant Chemoradiotherapies[Title/Abstract]) OR (Concomitant Radiochemotherapy[Title/Abstract]) OR (Concomitant Radiochemotherapies[Title/Abstract]) OR (Radiochemotherapies, Concomitant[Title/Abstract]) OR (Radiochemotherapy, Concomitant[Title/Abstract])	28134
#4	("Immune Checkpoint Inhibitors"[Mesh]) OR (Checkpoint Inhibitors, Immune[Title/Abstract]) OR (Immune Checkpoint Inhibitor[Title/Abstract]) OR (Checkpoint Inhibitor, Immune[Title/Abstract]) OR (Immune Checkpoint Blockers[Title/Abstract]) OR (Checkpoint Blockers, Immune[Title/Abstract]) OR (Immune Checkpoint Blockade[Title/Abstract]) OR (Checkpoint Blockade, Immune[Title/Abstract]) OR (Immune Checkpoint Inhibition[Title/Abstract]) OR (Checkpoint Inhibition, Immune[Title/Abstract]) OR (PD-L1 Inhibitors[Title/Abstract]) OR (PD L1	18274

	Inhibitors[Title/Abstract]) OR (PD-L1 Inhibitor[Title/Abstract]) OR (PD L1 Inhibitor[Title/Abstract]) OR (Programmed Death-Ligand 1 Inhibitors[Title/Abstract]) OR (Programmed Death Ligand 1 Inhibitors[Title/Abstract]) OR (PD-1-PD-L1 Blockade[Title/Abstract]) OR (Blockade, PD-1-PD-L1[Title/Abstract]) OR (PD 1 PD L1 Blockade[Title/Abstract]) OR (CTLA-4 Inhibitors[Title/Abstract]) OR (CTLA 4 Inhibitors[Title/Abstract]) OR (CTLA-4 Inhibitor[Title/Abstract]) OR (CTLA 4 Inhibitor[Title/Abstract]) OR (Cytotoxic T-Lymphocyte-Associated Protein 4 Inhibitors[Title/Abstract]) OR (Cytotoxic T Lymphocyte Associated Protein 4 Inhibitors[Title/Abstract]) OR (Cytotoxic T-Lymphocyte-Associated Protein 4 Inhibitor[Title/Abstract]) OR (Cytotoxic T Lymphocyte Associated Protein 4 Inhibitor[Title/Abstract]) OR (PD-1 Inhibitors[Title/Abstract]) OR (PD 1 Inhibitors[Title/Abstract]) OR (PD-1 Inhibitor[Title/Abstract]) OR (Inhibitor, PD-1[Title/Abstract]) OR (PD 1 Inhibitor[Title/Abstract]) OR (Programmed Cell Death Protein 1 Inhibitor[Title/Abstract]) OR (Programmed Cell Death Protein 1 Inhibitors[Title/Abstract])	
#5	#1 AND #2 AND #4	20
#6	#1 AND #3 AND #4	19
#7	#5 OR #6	22

Embase	Searching Strategy	Results
#1	'esophagus cancer'/exp OR 'cancer, esophagus':ti,ab,kw OR 'esophageal cancer':ti,ab,kw OR 'esophagus neoplasm':ti,ab,kw OR 'oesophageal cancer':ti,ab,kw OR 'oesophagus cancer':ti,ab,kw	91123
#2	'radiotherapy'/exp OR 'bioradiant therapy':ti,ab,kw OR 'bucky irradiation':ti,ab,kw OR 'bucky radiation':ti,ab,kw OR 'bucky radiotherapy':ti,ab,kw OR 'bucky ray':ti,ab,kw OR 'bucky ray radiation':ti,ab,kw OR 'bucky therapy':ti,ab,kw OR 'fractionated radiotherapy':ti,ab,kw OR 'hemibody irradiation':ti,ab,kw OR 'hypophysectomy, radiation':ti,ab,kw OR 'hypophysis irradiation':ti,ab,kw OR 'hypophysis radiation':ti,ab,kw OR 'irradiation therapy':ti,ab,kw OR 'irradiation treatment':ti,ab,kw OR 'irradiation, hypophysis':ti,ab,kw OR 'lymphatic irradiation':ti,ab,kw OR 'pituitary irradiation':ti,ab,kw OR 'radiation beam centration':ti,ab,kw OR 'radiation repair':ti,ab,kw OR 'radiation therapy':ti,ab,kw OR 'radiation treatment':ti,ab,kw OR 'radio therapy':ti,ab,kw OR 'radio treatment':ti,ab,kw OR 'radiohypophysectomy':ti,ab,kw OR 'radiology, therapeutic':ti,ab,kw OR 'radiotreatment':ti,ab,kw OR 'roentgen irradiation, therapeutic':ti,ab,kw OR 'roentgen therapy':ti,ab,kw OR 'roentgen treatment':ti,ab,kw OR 'rontgen therapy':ti,ab,kw OR 'therapeutic radiology':ti,ab,kw OR 'therapy, irradiation':ti,ab,kw OR 'therapy, radiation':ti,ab,kw OR 'therapy, roentgen':ti,ab,kw OR 'treatment, irradiation':ti,ab,kw OR 'treatment, radiation':ti,ab,kw OR 'treatment, roentgen':ti,ab,kw OR 'x radiotherapy':ti,ab,kw OR 'x ray therapy':ti,ab,kw OR 'x ray treatment':ti,ab,kw OR 'x-ray therapy':ti,ab,kw	686959
#3	'chemoradiotherapy'/exp OR 'chemoradiation':ti,ab,kw OR 'radiochemotherapy':ti,ab,kw	82323
#4	'immune checkpoint inhibitor'/exp OR 'immune checkpoint blocker':ti,ab,kw OR 'immune checkpoint inhibitors':ti,ab,kw	28608
#5	#1 AND #2 AND #4	148
#6	#1 AND #3 AND #4	82
#7	#5 OR #6	149

Scopus	Searching Strategy	Results
#1	TITLE-ABS-KEY ("esophageal neoplasm" OR "neoplasm, esophageal" OR "esophagus neoplasm" OR "esophagus neoplasms" OR "neoplasm, esophagus" OR "neoplasms, esophagus" OR "neoplasms, esophageal" OR "cancer of esophagus" OR "cancer of the esophagus" OR "esophagus cancer" OR "cancer, esophagus" OR "cancers, esophagus" OR "esophagus cancers" OR "esophageal cancer" OR "cancer, esophageal" OR "cancers, esophageal" OR "esophageal cancers" OR "oesophageal cancer" OR "oesophagus cancer" OR "cancer, oesophageal" OR "cancer, oesophagus" OR "oesophageal cancers" OR "oesophagus cancers" OR "cancers, oesophageal" OR "cancers, oesophagus")	80486
#2	TITLE-ABS-KEY ("radiotherapy" OR "radiotherapies" OR "radiation therapy" OR "radiation therapies" OR "therapies, radiation" OR "therapy, radiation" OR "radiation treatment" OR "radiation treatments" OR "treatment, radiation" OR "radiotherapy, targeted" OR "radiotherapies, targeted" OR "targeted radiotherapies" OR "targeted radiotherapy" OR "targeted radiation therapy" OR "radiation therapies, targeted" OR "targeted radiation therapies" OR "therapies, targeted radiation" OR "therapy, targeted radiation" OR "radiation therapy, targeted")	502773
#3	TITLE-ABS-KEY ("chemoradiotherapy" OR "chemoradiotherapies" OR "radiochemotherapy" OR "radiochemotherapies" OR "concurrent chemoradiotherapy" OR "chemoradiotherapies, concurrent" OR "chemoradiotherapy, concurrent" OR "concurrent chemoradiotherapies" OR "synchronous chemoradiotherapy" OR "chemoradiotherapies, synchronous" OR "chemoradiotherapy, synchronous" OR "synchronous chemoradiotherapies" OR "concurrent radiochemotherapy" OR "concurrent radiochemotherapies" OR "radiochemotherapies, concurrent" OR "radiochemotherapy, concurrent" OR "concomitant chemoradiotherapy" OR "chemoradiotherapies, concomitant" OR "chemoradiotherapy, concomitant" OR "concomitant chemoradiotherapies" OR "concomitant radiochemotherapy" OR "concomitant radiochemotherapies" OR "radiochemotherapies, concomitant" OR "radiochemotherapy, concomitant")	58193
#4	TITLE-ABS-KEY ("immune checkpoint inhibitors" OR "checkpoint inhibitors, immune" OR "immune checkpoint inhibitor" OR "checkpoint inhibitor, immune" OR "immune checkpoint blockers" OR "checkpoint blockers, immune" OR "immune checkpoint blockade" OR "checkpoint blockade, immune" OR "immune checkpoint inhibition" OR "checkpoint inhibition, immune" OR "pd-l1 inhibitors" OR "pd l1 inhibitors" OR "pd-l1 inhibitor" OR "pd l1 inhibitor" OR "programmed death-ligand 1 inhibitors" OR "programmed death ligand 1 inhibitors" OR "pd-1-pd-l1 blockade" OR "blockade, pd-1-pd-l1" OR "pd 1 pd l1 blockade" OR "ctla-4 inhibitors" OR "ctla 4 inhibitors" OR "ctla-4 inhibitor" OR "ctla 4 inhibitor" OR "cytotoxic t-lymphocyte-associated protein 4 inhibitors" OR "cytotoxic t lymphocyte associated protein 4 inhibitors" OR "cytotoxic t-lymphocyte-associated protein 4 inhibitor" OR "cytotoxic t lymphocyte associated protein 4 inhibitor" OR "pd-1 inhibitors" OR "pd 1 inhibitors" OR "pd-1 inhibitor" OR "inhibitor, pd-1" OR "pd 1 inhibitor" OR "programmed cell death protein 1 inhibitor" OR "programmed cell death protein 1 inhibitors")	30396
#5	#1 AND #2 AND #4	97
#6	#1 AND #3 AND #4	92
#7	#5 OR #6	164

Medline In-Process	Searching Strategy	Results
#1	exp Esophageal Neoplasms/	56849
#2	(cancer, esophageal or cancer, esophagus or cancer of esophagus or cancer of the esophagus or cancers, esophageal or cancers, esophagus or esophageal cancer or esophageal cancers or esophageal neoplasm or esophageal neoplasms or esophagus cancer or esophagus cancers or esophagus neoplasm or esophagus neoplasms or neoplasm, esophageal or neoplasm, esophagus or neoplasms, esophageal or neoplasms, esophagus).mp. [mp=title, abstract, original title, name of substance word, subject heading word, floating sub-heading word, keyword heading word, organism supplementary concept word, protocol supplementary concept word, rare disease supplementary concept word, unique identifier, synonyms]	59601
#3	exp Radiotherapy/	203117
#4	(radiation therapies or radiation therapies, targeted or radiation therapy or radiation therapy, targeted or radiation treatment or radiation treatments or radiotherapies or radiotherapies, targeted or radiotherapy or radiotherapy, targeted or targeted radiation therapies or targeted radiation therapy or targeted radiotherapies or targeted radiotherapy or therapies, radiation or therapies, targeted radiation or therapy, radiation or therapy, targeted radiation or treatment, radiation).mp. [mp=title, abstract, original title, name of substance word, subject heading word, floating sub-heading word, keyword heading word, organism supplementary concept word, protocol supplementary concept word, rare disease supplementary concept word, unique identifier, synonyms]	359307
#5	exp Chemoradiotherapy/	18977
#6	(chemoradiotherapies or chemoradiotherapies, concomitant or chemoradiotherapies, concurrent or chemoradiotherapies, synchronous or chemoradiotherapy or chemoradiotherapy, concomitant or chemoradiotherapy, concurrent or chemoradiotherapy, synchronous or concomitant chemoradiotherapies or concomitant chemoradiotherapy or concomitant radiochemotherapies or concomitant radiochemotherapy or concurrent chemoradiotherapies or concurrent chemoradiotherapy or concurrent radiochemotherapies or concurrent radiochemotherapy or radiochemotherapies or radiochemotherapies, concomitant or radiochemotherapies, concurrent or radiochemotherapy or radiochemotherapy, concomitant or radiochemotherapy, concurrent or synchronous chemoradiotherapies or synchronous chemoradiotherapy).mp. [mp=title, abstract, original title, name of substance word, subject heading word, floating sub-heading word, keyword heading word, organism supplementary concept word, protocol supplementary concept word, rare disease supplementary concept word, unique identifier, synonyms]	31346
#7	exp Immune Checkpoint Inhibitors/	14997
#8	(blockade, pd-1-pd-l1 or ctla 4 inhibitor or ctla 4 inhibitors or ctla-4 inhibitor or ctla-4 inhibitors or checkpoint blockade, immune or checkpoint blockers, immune or checkpoint inhibition, immune or checkpoint inhibitor, immune or checkpoint inhibitors, immune or cytotoxic t lymphocyte associated protein 4 inhibitor or cytotoxic t lymphocyte associated protein 4 inhibitors or cytotoxic t-lymphocyte-associated protein 4 inhibitor or cytotoxic t-lymphocyte-associated protein 4 inhibitors or immune checkpoint blockade or immune checkpoint blockers or immune checkpoint inhibition or immune checkpoint inhibitor or	19247

	immune checkpoint inhibitors or inhibitor, pd-1 or pd 1 inhibitor or pd 1 inhibitors or pd 1 pd l1 blockade or pd l1 inhibitor or pd l1 inhibitors or pd-1 inhibitor or pd-1 inhibitors or pd-1-pd-l1 blockade or pd-l1 inhibitor or pd-l1 inhibitors or programmed cell death protein 1 inhibitor or programmed cell death protein 1 inhibitors or programmed death ligand 1 inhibitors or programmed death-ligand 1 inhibitors).mp. [mp=title, abstract, original title, name of substance word, subject heading word, floating sub-heading word, keyword heading word, organism supplementary concept word, protocol supplementary concept word, rare disease supplementary concept word, unique identifier, synonyms]	
#9	#1 OR #2	59852
#10	#3 OR #4	389640
#11	#5 OR #6	31346
#12	#7 OR #8	26035
#13	#9 AND #10 AND #12	42
#14	#9 AND #11 AND #12	36
#15	#13 OR #14	54

Cochrane	Searching Strategy	Results
#1	MeSH descriptor: [Esophageal Neoplasms] explode all trees	1831
#2	(Esophageal Cancers):ti,ab,kw OR (Esophagus Cancer):ti,ab,kw OR (Cancer, Esophageal):ti,ab,kw OR (Esophagus Cancers):ti,ab,kw OR (Cancer of Esophagus):ti,ab,kw OR (Cancer of the Esophagus):ti,ab,kw OR (Cancers, Esophageal):ti,ab,kw OR (Cancer, Esophagus):ti,ab,kw OR (Cancers, Esophagus):ti,ab,kw OR (Esophageal Cancer):ti,ab,kw OR (Esophagus Neoplasms):ti,ab,kw OR (Neoplasm, Esophageal):ti,ab,kw OR (Neoplasm, Esophagus):ti,ab,kw OR (Neoplasms, Esophageal):ti,ab,kw OR (Esophagus Neoplasm):ti,ab,kw OR (Neoplasms, Esophagus):ti,ab,kw OR (Esophageal Neoplasm):ti,ab,kw	5756
#3	MeSH descriptor: [Radiotherapy] explode all trees	6669
#4	(Radiation Treatments):ti,ab,kw OR (Radiotherapies):ti,ab,kw OR (Radiation Therapies):ti,ab,kw OR (Therapy, Radiation):ti,ab,kw OR (Therapies, Radiation):ti,ab,kw OR (Treatment, Radiation):ti,ab,kw OR (Radiation Treatment):ti,ab,kw OR (Radiation Therapy):ti,ab,kw OR (Targeted Radiotherapy):ti,ab,kw OR (Targeted Radiotherapies):ti,ab,kw OR (Radiotherapies, Targeted):ti,ab,kw OR (Radiotherapy, Targeted):ti,ab,kw OR (Radiation Therapy, Targeted):ti,ab,kw OR (Radiation Therapies, Targeted):ti,ab,kw OR (Therapy, Targeted Radiation):ti,ab,kw OR (Targeted Radiation Therapies):ti,ab,kw OR (Therapies, Targeted Radiation):ti,ab,kw OR (Targeted Radiation Therapy):ti,ab,kw	25534
#5	MeSH descriptor: [Chemoradiotherapy] explode all trees	1179
#6	(Radiochemotherapy, Concurrent):ti,ab,kw OR (Concurrent Radiochemotherapies):ti,ab,kw OR (Synchronous Chemoradiotherapy):ti,ab,kw OR (Radiochemotherapies, Concurrent):ti,ab,kw OR (Concomitant Radiochemotherapy):ti,ab,kw OR (Chemoradiotherapy, Concurrent):ti,ab,kw OR (Chemoradiotherapies, Synchronous):ti,ab,kw OR (Chemoradiotherapy, Synchronous):ti,ab,kw OR (Concomitant Radiochemotherapies):ti,ab,kw OR (Concurrent Chemoradiotherapy):ti,ab,kw OR (Chemoradiotherapy, Concomitant):ti,ab,kw OR (Concurrent Radiochemotherapy):ti,ab,kw OR (Chemoradiotherapies, Concomitant):ti,ab,kw OR (Concomitant Chemoradiotherapy):ti,ab,kw OR (Concomitant Chemoradiotherapies):ti,ab,kw OR (Radiochemotherapy, Concomitant):ti,ab,kw OR (Concurrent Chemoradiotherapies):ti,ab,kw OR (Chemoradiotherapies, Concurrent):ti,ab,kw OR (Radiochemotherapies, Concomitant):ti,ab,kw OR (Synchronous Chemoradiotherapies):ti,ab,kw OR (Chemoradiotherapies):ti,ab,kw OR (Radiochemotherapies):ti,ab,kw OR (Radiochemotherapy):ti,ab,kw	4229
#7	MeSH descriptor: [Immune Checkpoint Inhibitors] explode all trees	78
#8	(Immune Checkpoint Inhibition):ti,ab,kw OR (Checkpoint Blockade, Immune):ti,ab,kw OR (Immune Checkpoint Blockade):ti,ab,kw OR (Checkpoint Inhibition, Immune):ti,ab,kw OR (Programmed Death-Ligand 1 Inhibitors):ti,ab,kw OR (PD-L1 Inhibitors):ti,ab,kw OR (Programmed Death Ligand 1 Inhibitors):ti,ab,kw OR (PD L1 Inhibitor):ti,ab,kw OR (PD-L1 Inhibitor):ti,ab,kw OR (PD L1 Inhibitors):ti,ab,kw OR (PD-1 Inhibitor):ti,ab,kw OR (Programmed Cell Death Protein 1 Inhibitor):ti,ab,kw OR (PD 1 Inhibitor):ti,ab,kw OR (Programmed Cell Death Protein 1 Inhibitors):ti,ab,kw OR (PD 1 Inhibitors):ti,ab,kw OR (Inhibitor, PD-1):ti,ab,kw OR (PD-1 Inhibitors):ti,ab,kw OR	6735

	(Cytotoxic T Lymphocyte Associated Protein 4 Inhibitor):ti,ab,kw OR (Cytotoxic T-Lymphocyte-Associated Protein 4 Inhibitor):ti,ab,kw OR (PD-1 PD-L1 Blockade):ti,ab,kw OR (PD 1 PD L1 Blockade):ti,ab,kw OR (Blockade, PD-1 PD-L1):ti,ab,kw OR (Checkpoint Blockers, Immune):ti,ab,kw OR (Checkpoint Inhibitors, Immune):ti,ab,kw OR (Checkpoint Inhibitor, Immune):ti,ab,kw OR (Immune Checkpoint Blockers):ti,ab,kw OR (Immune Checkpoint Inhibitor):ti,ab,kw	
#9	(#1 or #2) and (#3 or #4) and (#7 or #8)	15
#10	(#1 or #2) and (#5 or #6) and (#7 or #8)	15
#11	#9 or #10	20

ASCO	Searching Strategy	Results
#1	(Keywords:"esophageal" OR Keywords:"esophagus" OR Keywords:"oesophageal" OR Keywords:"oesophagus" OR Keywords:"gastroesophageal" OR Keywords:"oesophagogastric" OR Keywords:"esophagogastric") AND (Keywords:"Immunotherapy" OR Keywords:"Immunotherapies" OR Keywords:"Ipilimumab" OR Keywords:"PD-1" OR Keywords:"PD-L1" OR Keywords:"ICI" OR Keywords:"Nivolumab" OR Keywords:"Opdivo" OR Keywords:"pembrolizumab" OR Keywords:"lambrolizumab" OR Keywords:"Keytruda" OR Keywords:"sintilimab" OR Keywords:"camrelizumab" OR Keywords:"Cemiplimab" OR Keywords:"toripalimab" OR Keywords:"Tislelizumab" OR Keywords:"Atezolizumab" OR Keywords:"Tecentriq" OR Keywords:"durvalumab" OR Keywords:"Imfinzi" OR Keywords:"avelumab" OR Keywords:"bavencio" OR Keywords:"Sugemalimab" OR Keywords:"checkpoint" OR Keywords:"Programmed") AND (Keywords:"radiotherapy" OR Keywords:"radiation" OR Keywords:"irradiation" OR Keywords:"chemoradiation" OR Keywords:"chemoradiotherapy")<br>Filters: ASCO ANNUAL MEETING; ASCO GASTROINTESTINAL CANCERS SYMPOSIUM;<br>Filters: Research	10

ESMO	Searching Strategy	Results
#1	(esophageal OR esophagus OR oesophageal OR oesophagus OR gastroesophageal OR oesophagogastric OR esophagogastric) AND (Immunotherapy OR Immunotherapies OR Ipilimumab OR PD-1 OR PD-L1 OR ICI OR Nivolumab OR Opdivo OR pembrolizumab OR lambrolizumab OR Keytruda OR sintilimab OR camrelizumab OR Cemiplimab OR toripalimab OR Tislelizumab OR Atezolizumab OR Tecentriq OR durvalumab OR Imfinzi OR avelumab OR bavencio OR Sugemalimab OR checkpoint OR Programmed) AND (radiotherapy OR radiation OR irradiation OR chemoradiation OR chemoradiotherapy)<br>Filters: Abstract	75

AACR	Searching Strategy	Results
#1	(esophageal) AND (radiotherapy) AND (Immunotherapy OR PD-1 OR PD-L1 OR checkpoint)<br>Filters: Cancer Research<br>published: 01/01/2012 to 08/21/2022	31

ASTRO	Searching Strategy	Results
#1	AbstractTitleKeywordFilterField:"esophageal" OR AbstractTitleKeywordFilterField:"esophagus" OR AbstractTitleKeywordFilterField:"oesophageal" OR AbstractTitleKeywordFilterField:"oesophagus" OR AbstractTitleKeywordFilterField:"gastroesophageal" OR AbstractTitleKeywordFilterField:"oesophagogastric" OR AbstractTitleKeywordFilterField:"esophagogastric"	1738
#2	AbstractTitleKeywordFilterField:"Immunotherapy" OR AbstractTitleKeywordFilterField:"Immunotherapies" OR AbstractTitleKeywordFilterField:"Ipilimumab" OR AbstractTitleKeywordFilterField:"PD-1" OR AbstractTitleKeywordFilterField:"PD-L1" OR AbstractTitleKeywordFilterField:"ICI" OR AbstractTitleKeywordFilterField:"Nivolumab" OR AbstractTitleKeywordFilterField:"Opdivo" OR AbstractTitleKeywordFilterField:"pembrolizumab" OR AbstractTitleKeywordFilterField:"lambrolizumab" OR AbstractTitleKeywordFilterField:"Keytruda" OR AbstractTitleKeywordFilterField:"sintilimab" OR AbstractTitleKeywordFilterField:"camrelizumab" OR AbstractTitleKeywordFilterField:"Cemiplimab" OR AbstractTitleKeywordFilterField:"toripalimab" OR AbstractTitleKeywordFilterField:"Tislelizumab" OR AbstractTitleKeywordFilterField:"Atezolizumab" OR AbstractTitleKeywordFilterField:"Tecentriq" OR AbstractTitleKeywordFilterField:"durvalumab" OR AbstractTitleKeywordFilterField:"Imfinzi" OR AbstractTitleKeywordFilterField:"avelumab" OR AbstractTitleKeywordFilterField:"bavencio" OR AbstractTitleKeywordFilterField:"Sugemalimab" OR AbstractTitleKeywordFilterField:"checkpoint" OR AbstractTitleKeywordFilterField:"Programmed"	849
#3	AbstractTitleKeywordFilterField:"radiotherapy" OR AbstractTitleKeywordFilterField:"radiation" OR AbstractTitleKeywordFilterField:"irradiation" OR AbstractTitleKeywordFilterField:"chemoradiation" OR AbstractTitleKeywordFilterField:"chemoradiotherapy"	56123
#4	#1 AND #2 AND #3	20

Supplementary Table 2. Baseline characteristics of clinical trials included (n=14)

Trials identifier	First Author	Year	Publication Format	Phase	Study design	Clinical Stage	Enrollment	Median age (Range)	Gender distribution (male/female)	Histology	Drug	Cycle	Line of therapy	Sequence	Radiotherapy
ChiCTR2000040533	Chen. Y.	2022	Conference Abstract	II	Cohort study	IVB	34	60（NA）	85.3%/14.7%	Squamous 100%	camrelizumab 200mg. q3w	Unlimited	Unlimited	Concurrent	Conventional 30-50Gy
NCT03490292	Uboha. N.V.	2022	Conference Abstract	I/II	Cohort study	II-III	22	64（NA）	90.9%/9.1%	Squamous 14% /Adenocarcinoma 86%	avelumab 10mg/kg. q2w	3 + 6 Cycles	Neoadjuvant + Consolidation	Sequential	Conventional 41.4Gy
NCT03792347	Li. C.	2021	Article	I	Cohort study	II-IVA	20	62（42-66）	95%/5%	Squamous 100%	pembrolizumab 2mg/kg. q3w	2 Cycles	Neoadjuvant	Concurrent	Conventional 41.4Gy
NCT02639065	Mamdani. H.	2021	Article	II	Cohort study	IIB-IVA	37	61（43-73）	97.3%/2.7%	Adenocarcinoma 100%	durvalumab 1500mg. q4w	13 Cycles	Consolidation	Sequential	NA
NCT02520453	Park. S.	2022	Artical	II	RCT	II-III	45	64（39-76）	96%/4%	Squamous 100%	durvalumab 20mg/kg. q4w	Up to 1 year	Consolidation	Sequential	Conventional 44Gy
NCT03087864	Van Den Ende. T.	2021	Artical	II	Cohort study	II-III	40	63（40-75）	87.5%/12.5%	Adenocarcinoma 100%	atezolizumab 1200mg. q3w	5 Cycles	Neoadjuvant	Concurrent	Conventional 41.4Gy
NCT04286958	Wang. J.	2021	Conference Abstract	II	Cohort study	II-IVA	11	NA	NA	Squamous 100%	camrelizumab 200mg. q2w	Up to 1 year	Consolidation	Sequential	Conventional 50-60Gy
ChiCTR1900022282	Sun. H.	2021	Conference Abstract	I	Cohort study	II-III	22	60（48-70）	77%/23%	Squamous 100%	toripalimab 240mg. q3w	4 Cycles	Neoadjuvant	Concurrent	Conventional 41.4Gy
NA	Qi. W.X.	2020	Conference Abstract	I	Cohort study	III-IVA	20	61.2（39-66）	95%/5%	Squamous 100%	pembrolizumab 2mg/kg. q3w	2 Cycles	Neoadjuvant	Concurrent	Conventional 41.4Gy
NCT02743494	Kelly. R.J.	2021	Article	III	RCT	II-III	532	62（26-82）	84%/16%	Squamous 29% /Adenocarcinoma 71%	nivolumab 240mg. q2w for 16 weeks, followed by nivolumab 480mg. Q4w	Up to 1 year	Adjuvant or Consolidation	Sequential	Conventional 41.4-60Gy
NCT03671265	Zhang. W.	2021	Article	I	Cohort study	II-IVA	20	64（56-74）	90%/10%	Squamous 100%	camrelizumab 200mg. q2w	16 Cycles	First-line + Consolidation	Concurrent	Conventional 60Gy
NCT03222440	Pang Q.	2018	Conference Abstract	I	Cohort study	II-IVA	20	NA	NA	Squamous 100%	camrelizumab 200mg. q2w	16 Cycles	First-line + Consolidation	Concurrent	Conventional 60Gy
NA	Jing. Z.	2018	Conference Abstract	I	Cohort study	II-IVA	16	61（35-70）	NA	Squamous 100%	camrelizumab 200mg. q2w	5 Cycles	First-line	Concurrent	Conventional 60Gy
NCT02844075	Lee. S.	2019	Conference Abstract	II	Cohort study	Ib-III	28	60（NA）	NA	Squamous 100%	pembrolizumab 200mg. q3w	Up to 2 years	Neoadjuvant + Consolidation	Concurrent	Conventional 44.1Gy

Supplementary Table 3. Main characteristics of ICI arms included in the meta-analysis for AEs comparison (n=14)

First Author	Drug type	Median follow-up (range), months	No. of pts in safety Analysis	All-grade trAEs, No.	Grade 3-4 trAEs, No.	Grade 5 trAEs, No.	Causes of treatment-related deaths	trAEs leading to ICI discontinuation, No.
Chen. Y.	PD-1	14.3(5.6-25.3)	34	32	13	0	None	NA
Uboha. N.V.	PD-L1	NA	22	NA	NA	0	None	1
Li. C.	PD-1	6.6(2.2-12.3)	20	20	13	1	Oesophageal haemorrhage	1
Mamdani. H.	PD-L1	17.7(1.7-24.3)	37	30	10	0	None	8
Park. S.	PD-L1	38.7(36.8-43.3)	45	26	3	0	None	3
Van Den Ende. T.	PD-L1	24（NA）	40	38	16	1	Pulmonary embolus	3
Wang. J.	PD-1	6.9（NA）	11	NA	1	0	None	0
Sun. H.	PD-1	NA	22	15	1	0	None	1
Qi. W.X.	PD-1	NA	20	20	NA	1	GI haemorrhage	0
Kelly. R.J.	PD-1	24.4（6.2-44.9）	532	379	74	0	None	48
Zhang. W.	PD-1	23.7（21.9-25.4）	20	20	9	0	None	1
Pang Q.	PD-1	NA	18	NA	1	0	None	0
Jing. Z.	PD-1	3（NA）	14	6	0	0	None	0
Lee. S.	PD-1	12.4（NA）	28	NA	NA	NA	NA	NA

Supplementary Table 4. Risk of bias and quality assessment of studies included using NOS (n=14)

Study	Selection 1	Selection 2	Selection 3	Selection 4	Comparability A	Comparability B	Outcome 1	Outcome 2	Outcome 3	Scores
	Representativeness of the exposed cohort	Selection of non-exposed cohort	Ascertainment of exposure	Demonstration that outcome of interest was not present at start of study	Comparability of cohorts on the basis of the design or analysis	Study controls for any additional factors	Assessment of outcome	Follow-up long enough for outcome to occur	Adequacy of follow-up of cohorts	Total
Chen 2022	*	-	*	*	-	-	*	*	*	6
Uboha 2022	*	-	*	*	-	-	*	*	*	6
Li 2021	*	-	*	*	-	-	*	*	*	6
Mamda 2021	*	-	*	*	-	-	*	*	*	6
Park 2022	*	*	*	*	*	*	*	*	*	9
Van 2021	*	-	*	*	-	-	*	*	*	6
Wang 2021	*	-	*	*	-	-	*	*	*	6
Sun 2021	*	-	*	*	-	-	*	*	*	6
Qi 2020	*	-	*	*	-	-	*	*	*	6
Kelly 2021	*	*	*	*	*	*	*	*	*	9
Zhang 2021	*	-	*	*	-	-	*	*	*	6
Pang 2018	*	-	*	*	-	-	*	*	*	6
Jing 2018	*	-	*	*	-	-	*	*	*	6
Lee 2019	*	-	*	*	-	-	*	*	*	6

Supplementary Table 5. Risk of bias and quality assessment of studies included using MINORS (n=14)

Study	Methodological items for non-randomized studies								Additional criteria in the case of comparative study				Total scores
	A clearly stated aim	Inclusion of consecutive patients	Prospective collection of data	Endpoints appropriate to the aim of the study	Unbiased assessment of the study endpoint	Follow-up period appropriate to the aim of the study	Loss to follow up less than 5%	Prospective calculation of the study size	An adequate control group	Contemporary groups	Baseline equivalence of groups	Adequate statistical analyses	
Chen 2022	2	2	2	2	0	2	2	2	-	-	-	-	14
Uboha 2022	2	2	2	2	0	0	2	2	-	-	-	-	12
Li 2021	2	2	2	2	0	1	2	2	-	-	-	-	13
Mamda 2021	2	2	2	2	0	2	2	2	-	-	-	-	14
Park 2022	2	2	2	2	2	2	2	2	2	2	2	2	24
Van 2021	2	2	2	2	0	2	2	2	-	-	-	-	14
Wang 2021	2	2	2	2	0	1	2	2	-	-	-	-	13
Sun 2021	2	2	2	2	0	0	2	2	-	-	-	-	12
Qi 2020	2	2	2	2	0	0	2	2	-	-	-	-	12
Kelly 2021	2	2	2	2	2	2	2	2	2	2	2	2	24
Zhang 2021	2	2	2	2	0	2	2	2	-	-	-	-	14
Pang 2018	2	2	2	2	0	0	2	2	-	-	-	-	12
Jing 2018	2	2	2	2	0	1	2	2	-	-	-	-	13
Lee 2019	2	2	2	2	0	2	2	2	-	-	-	-	14
