# Supplementary figures and images for: Toxicity profile of combined immune checkpoint inhibitors and thoracic radiotherapy in esophageal cancer: A meta-analysis and systematic review
Source: Front Immunol. 2022 Nov 10;13:1039020. doi: 10.3389/fimmu.2022.1039020 (PMC9685562; doi:10.3389/fimmu.2022.1039020)

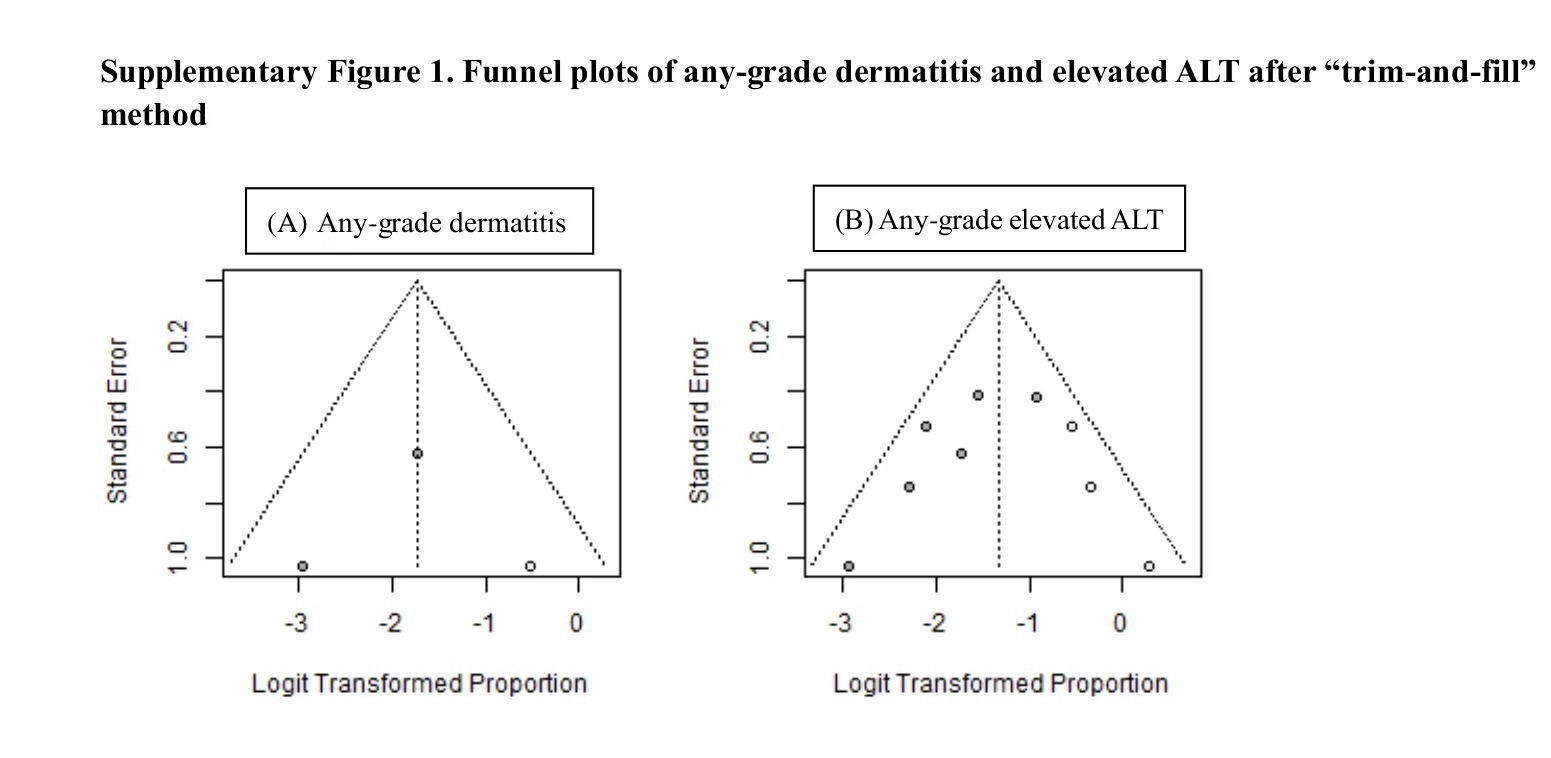

Supplement: Supplementary file 2 [file Image_1.jpeg]

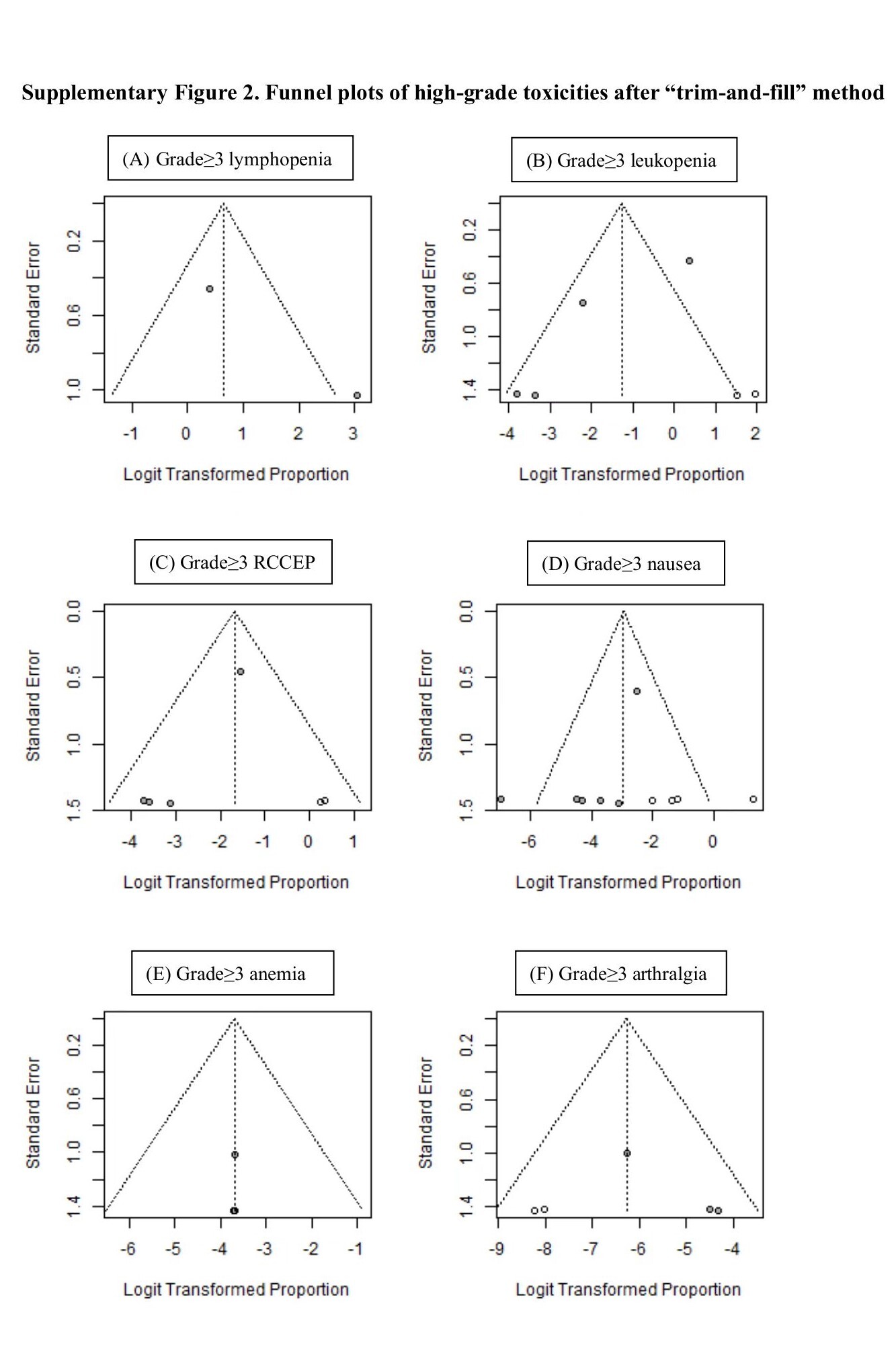

Supplement: Supplementary file 3 [file Image_2.jpeg]
